# SMOC approach for total knee arthroplasty in valgus knees

**DOI:** 10.1186/s13018-022-03002-x

**Published:** 2022-02-22

**Authors:** Tao Li, Yikai Liu, Chenkai Li, Haining Zhang

**Affiliations:** Department of Joint Surgery, The Affiliated Hospital of Qingdao University, Qingdao, 266000 Shandong China

**Keywords:** Total knee arthroplasty, Valgus, Vastus medialis oblique, Surgical approach

## Abstract

**Objective:**

This study was performed to compare clinical outcomes among patients with valgus knees undergoing total knee arthroplasty via the medial parapatellar approach and the subvastus with minimal oblique cut approach.

**Methods:**

A total of 232 patients (246 knees) undergoing total knee arthroplasty between December 2014 and December 2016 were retrospectively included in the investigation. The study population consisted of 120 patients (128 knees; 32 men and 88 women) with a mean age of 62.43 ± 8.12 years treated via the medial parapatellar approach, and 112 patients (118 knees; 30 men and 82 women with a mean age of 63.15 ± 7.83 years) treated via the subvastus with minimal oblique cut approach. Nine preoperative parameters (number of patients, sex, age, body mass index, number of knees, valgus angle, visual analogue scale score, range of motion, Hospital for Special Surgery score), five perioperative parameters (operative time, amount of drainage, Visual analogue scale score at 24 h after the operation, time to straight leg raising, radiological alignment), and two postoperative parameters (range of motion, Hospital for Special Surgery score) were assessed at 1 day, 1 week, 6 weeks, 8 weeks and 1 year after the operation, along with postoperative complications.

**Results:**

There were no significant differences in the nine preoperative parameters between the two groups. The subvastus with minimal oblique cut group had a longer operative time, while the parapatellar approach group showed more drainage and a higher mean Visual analogue scale score. Compared to the medial parapatellar group, the subvastus with minimal oblique cut group had a shorter time to straight leg raising. There were no differences in radiological alignment between the two groups. The groups showed similar range of motion and Hospital for Special Surgery scores at 8 weeks and 1 year, but both were higher in the subvastus with minimal oblique cut group at 1 day, 1 week and 6 weeks. During postoperative follow-up, postoperative subluxation of the patella occurred in five cases in the medial parapatellar group. Neither group showed any instability, recurrent valgus deformity or radiographic loosening.

**Conclusion:**

The subvastus with minimal oblique cut approach provides excellent early recovery for total knee arthroplasty of valgus knees with no increase in complications.

## Background

Total knee arthroplasty (TKA) was first performed in 1968 and is the only effective treatment for patients with end-stage osteoarthritis of the knee [1, 2]. TKA is the most effective surgical procedure for relieving pain and improving function in patients with advanced arthritis of the knee [3, 4]. The utilisation of TKA has increased markedly over the past several decades, and an estimated 3.5 million TKAs will be performed annually by 2030 [5].

The standard medial parapatellar (MP) approach most widely used in varus knees has been reported to show unsatisfactory results, including incomplete axis restoration, changes in the joint line, constrained implants, underlying instability and poor clinical outcomes [6–9]. Recently, the subvastus approach without tibial medial collateral ligament release [10], has also been used in the treatment of type I and II valgus knees. The subvastus with minimal oblique cut (SMOC) approach, combined with the subvastus with limited parapatellar approach, protects the vastus medialis oblique (VMO) and patellar tendon from damage and excessive release during exposure, expands the indications for minimally invasive surgery (MIS), and reduces the requirement for specialised or custom instruments. The SMOC approach has been used in TKA for treatment of varus deformity, but has seldom been applied in TKA for valgus knees.

Therefore, this study was performed to evaluate the clinical and radiological outcomes of the SMOC approach in valgus knees in terms of functional recovery, tibiofemoral alignment and postoperative complications.

## Materials and methods

### Inclusion and exclusion criteria

The inclusion criteria were as follows: primary TKA performed by one senior surgeon (Z.H.) between December 2014 and December 2016, using the MP or SMOC approach, and posterior stabilised implants.

The exclusion criteria were varus knee and use of a special prosthesis, such as a constrained knee implant.

### Surgical procedures

Similar perioperative procedures were applied in both approaches. Patients were anaesthetised via femoral nerve block to optimise muscle relaxation, and all skin incisions were performed with the knee in 60° flexion. The tourniquet was released just before closure. Periarticular injection of a multimodal drug cocktail consisting of an analgesic and opioid was used for pain relief following TKA. Antibiotics were only used once before surgery and twice after TKA. Celecoxib was used for analgesia, and supplementary analgesia with pethidine hydrochloride was added if necessary. Rivaroxaban was used once daily for 14 days, for prevention of deep venous thrombosis. Drainage was removed 24 h postoperatively. Ankle flexion and extension exercises, along with straight leg raising (SLR), were encouraged from the day of the operation. Continuous passive motion was utilised from day 1 postoperatively.

The MP approach was essentially performed according to the original description by von Langenbeck [11]. A standard longitudinal midline skin incision (15–20 cm) was made, and the parapatellar retinacular incision was extended proximally along the length of the quadriceps tendon, leaving a 3–4-mm cuff of tendon on the VMO for later closure. It was extended by 3–4 cm on to the anteromedial surface of the tibia along the medial border of the patellar tendon, by following the course of the medial side of the patella [12]. The mobile upper aspect of the skin incision was used to expose the proximal portion of the quadriceps tendon. The patella was everted to allow release of the lateral patellofemoral plica if necessary, and conventional osteotomy was used in medial arthrotomy.

**Fig. 1:**
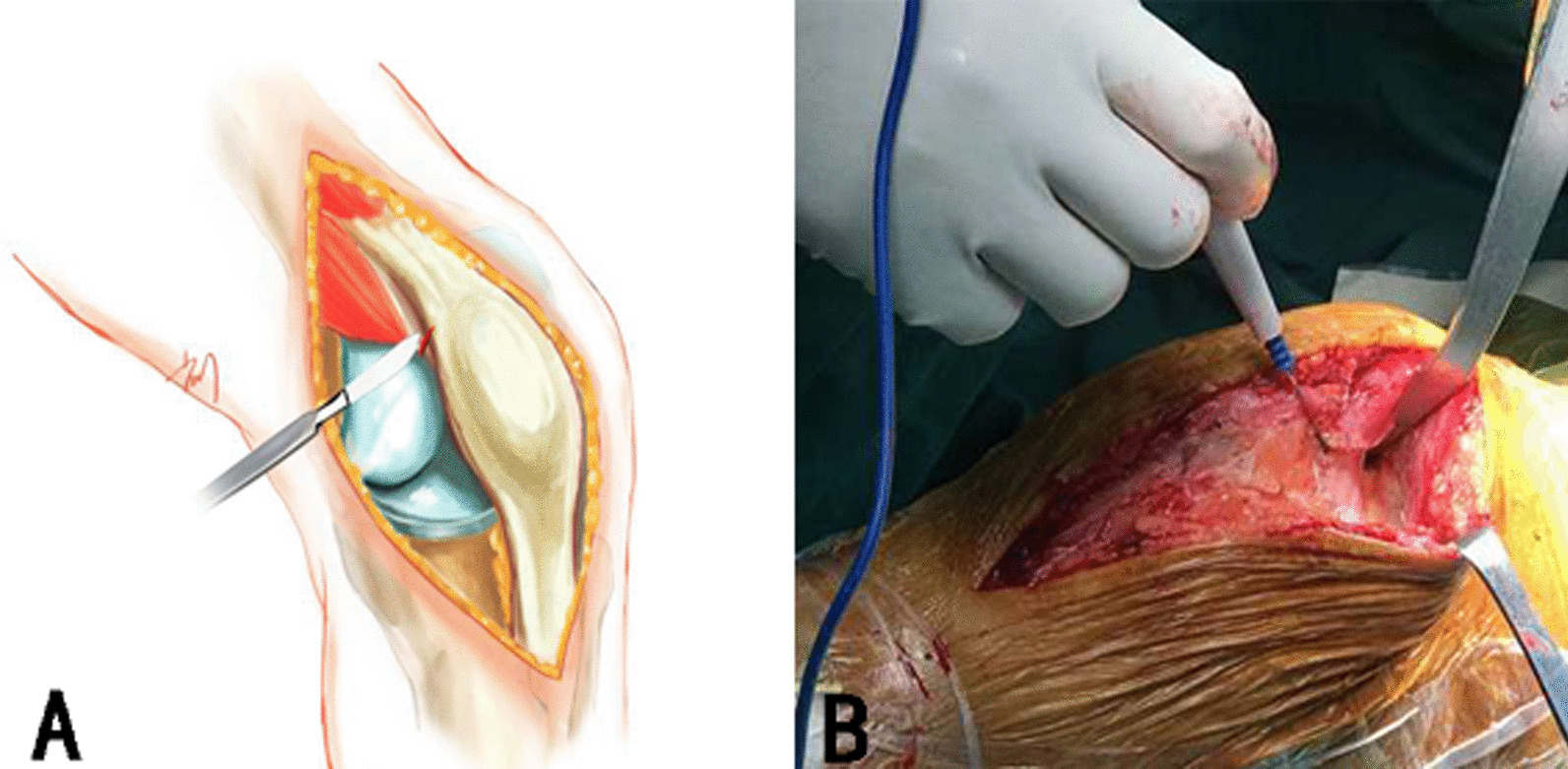
The details of SMOC approach

In the SMOC group, a midline incision 7–12 cm in length was made from the superior pole of the patella to 2 cm below the joint line medial to the tibial tubercle [13]. The medial soft tissue sleeve was released to expose the VMO. After opening the myolemma, the VMO was released from the intermuscular septum and a Hoffmann retractor was inserted below the VMO up to the lateral sulcus. The VMO was then released proximally (within 5 cm) and elevated with the Hoffmann retractor. A midline incision was made on the suprapatellar capsule and cut down to the skin incision level, parallel with the patellar tendon. Throughout the operation, the patella was retracted rather than everted. In some cases, such as large muscular patients and patients with severe deformity, a far distal VMO insertion or short patellar tendon (leading to exposure difficulty), an oblique cut was performed. Beginning from the medial superior corner of the patella, we left 5 mm of tendon tissue for quadriceps insertion, and an “oblique laterosuperior cut” 0–2 cm in length was made at an angle of 45° (Fig. 1). Medial ligament release was not performed.

### Data collection

A total of 246 knees in 232 patients with valgus deformity were divided into the MP group (120 patients, 128 knees; 32 men and 88 women aged 62.43 ± 8.12 years) and SMOC group (112 patients, 118 knees; 30 men and 82 women aged 63.15 ± 7.83 years). The MP group included 76 cases of type I valgus and 52 of type II valgus, while the SMOC group included 64 cases of type I valgus and 48 of type II valgus. A cemented, posterior-stabilised prosthesis (Zimmer, Warsaw, IN, USA) was used for all knees. To evaluate the short-term clinical outcomes associated with utilisation of the MP and SMOC approaches, nine preoperative parameters (number of patients, sex, age, body mass index, number of knees, valgus angle, visual analogue scale [VAS] score, range of motion [ROM], Hospital for Special Surgery [HSS] score), five perioperative parameters (operative time, amount of drainage, VAS at 24 h after the operation, time to SLR, radiological alignment), two postoperative parameters (ROM, HSS score) and postoperative complications were assessed at 1 day, 1 week, 6 weeks, 8 weeks and 1 year after the operation.

### Statistical analysis

Statistical analyses were performed using SPSS 19.0 software (SPSS Inc., Chicago, IL, USA). Continuous data are expressed as the mean ± standard deviation (SD). When the parametric test assumptions were met, Student’s *t* test or the Mann–Whitney U test was used for comparing independent groups in terms of numerical variables. The *χ*^2^ test was used for analysing categorical variables. The *α* level was determined as *P* < 0.05 (two-tailed comparison).

## Results

### Preoperative parameters

The data of the nine preoperative parameters are listed in Table 1. There were no significant differences in the number of patients, sex, age, body mass index, number of knees, valgus angle, VAS score, ROM or HSS score between the MP and SMOC groups (all *P* > 0.05, *χ*^2^ test or Student’s *t* test).

**Table 1:** Clinical data of individual patients included in the study

Variables	SMOC	MP	*P* value
Age (Y)	63.15 ± 7.83	62.43 ± 8.12	0.493
Gender	112	120	> 0.9999
Male	30	32	
Female	82	88	
BMI (kg/m^2^)	24.05 ± 5.64	23.21 ± 5.33	0.2447
valgus angle	18 ± 10.24	20 ± 11.34	0.1595
Type (I: II)*	64:48	76:52	0.7932
VAS score	5.33 ± 1.28	5.46 ± 1.06	0.4022
ROM (°)	100.2 ± 14.2	102.1 ± 16.33	0.3445
HSS	35.25 ± 8.26	36.27 ± 8.13	0.3446

^*^The classification is made according to the Ranawat valgus knee classification

### Perioperative parameters

The data of the five perioperative parameters are provided in Table 2 (results of Student’s *t* test). The operative time was longer in the SMOC than MP group, but the difference was not significant (76.20 ± 13.24 vs. 73.65 ± 12.68 min, *P* = 0.1075). The MP group had a higher mean amount of drainage, higher VAS score at 24 h after the operation, and longer time to SLR than the SMOC group (168.34 ± 40.21 vs. 96.57 ± 46.28 ml, 5.33 ± 1.28 vs. 4.62 ± 1.06, and 2.45 ± 1.33 vs. 1.12 ± 0.78 d, respectively, all *P* < 0.001). The average femorotibial angle was corrected from 18° ± 10.24° preoperatively to 5.5° ± 3.11° postoperatively in the MP group, and from 20° ± 11.34° preoperatively to 4.8° ± 2.86° postoperatively in the SMOC group.

**Table 2:** Perioperative parameters

Variables	SMOC	MP	*P* value
Operative time (min)	76.20 ± 13.24	73.65 ± 12.68	0.1354
Amount of drainage (ml)	96.57 ± 46.28	168.34 ± 40.21	< 0.0001
VAS score at 24 h after operation	4.62 ± 1.06	5.33 ± 2.28	< 0.0001
Time of SLR (d)	1.12 ± 0.78	2.45 ± 1.33	< 0.0001
Radiological alignment (°)	4.8 ± 2.86	5.5 ± 3.11	0.0763

### Postoperative parameters

The data of the two postoperative parameters are provided in Tables 3 and 4 (Student’s *t* test and Mann–Whitney U-test results). The SMOC group had a higher mean ROM than the MP group at 1 day, 1 week and 6 weeks (80° ± 10.6° vs. 72° ± 9.34°, 97.68° ± 6.23° vs. 84.23° ± 10.3° and 105.2° ± 5.28° vs. 100° ± 10.15°, respectively, all *P* < 0.001). ROM at 8 weeks and 1 year were similar in both groups (107.3 ± 3.6 vs. 108.1 ± 2.48 and 109.2 ± 1.04 vs. 109.1 ± 1.14, respectively, all *P* > 0.05). HSS scores were higher at 1 day, 1 week and 6 weeks in the SMOC than MP group (40.82 ± 5.34 vs. 38.45 ± 8.13, 52.34 ± 5.75 vs. 42.85 ± 5.12 and 80.28 ± 5.34 vs. 78.2 ± 6.38, respectively, all *P* < 0.001). There were no differences in HSS scores at 8 weeks or 1 year between the two groups MP (88.3 ± 5.56 vs. 87.62 ± 4.71 and 92.7 ± 2.34 vs. 92.4 ± 1.78, respectively, all *P* > 0.05).

**Table 3:** Postoperative ROM

Group	1 day	1 week	6 weeks	8 weeks	1 year
SMOC	80 ± 10.6	97.68 ± 6.23	105.2 ± 5.28	108.1 ± 2.48	109.2 ± 1.04
MP	72.44 ± 9.34	84.23 ± 10.3	100 ± 10.15	107.3 ± 3.6	109.1 ± 1.14
*P* value	< 0.0001	< 0.0001	< 0.0001	0.0487	0.4869

**Table 4:** postoperative HSS

Group	1 day	1 week	6 weeks	8 weeks	1 year
SMOC	40.82 ± 5.34	52.34 ± 5.75	80.28 ± 5.34	88.3 ± 5.56	92.7 ± 2.34
MP	38.45 ± 8.13	42.85 ± 5.12	78.2 ± 6.38	87.62 ± 4.71	92.4 ± 1.78
*P* value	0.0098	< 0.0001	0.0078	0.3148	0.1864

**Fig. 2:**
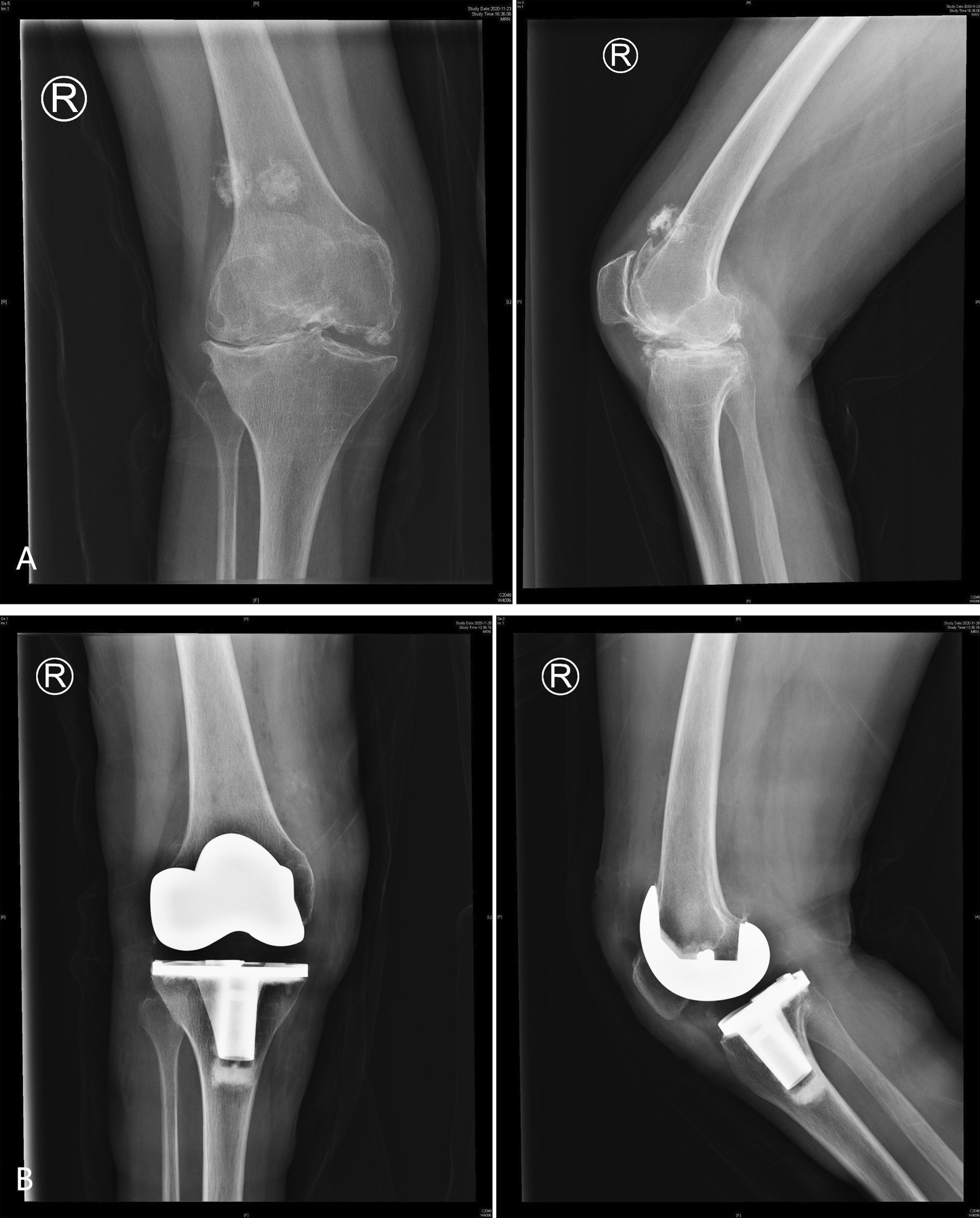
The anteroposterior and lateral knee radiographs of right knee before and after the operation. **A** preoperative valgus knee with osteophyte; **B** postoperative knee status

**Fig. 3:**
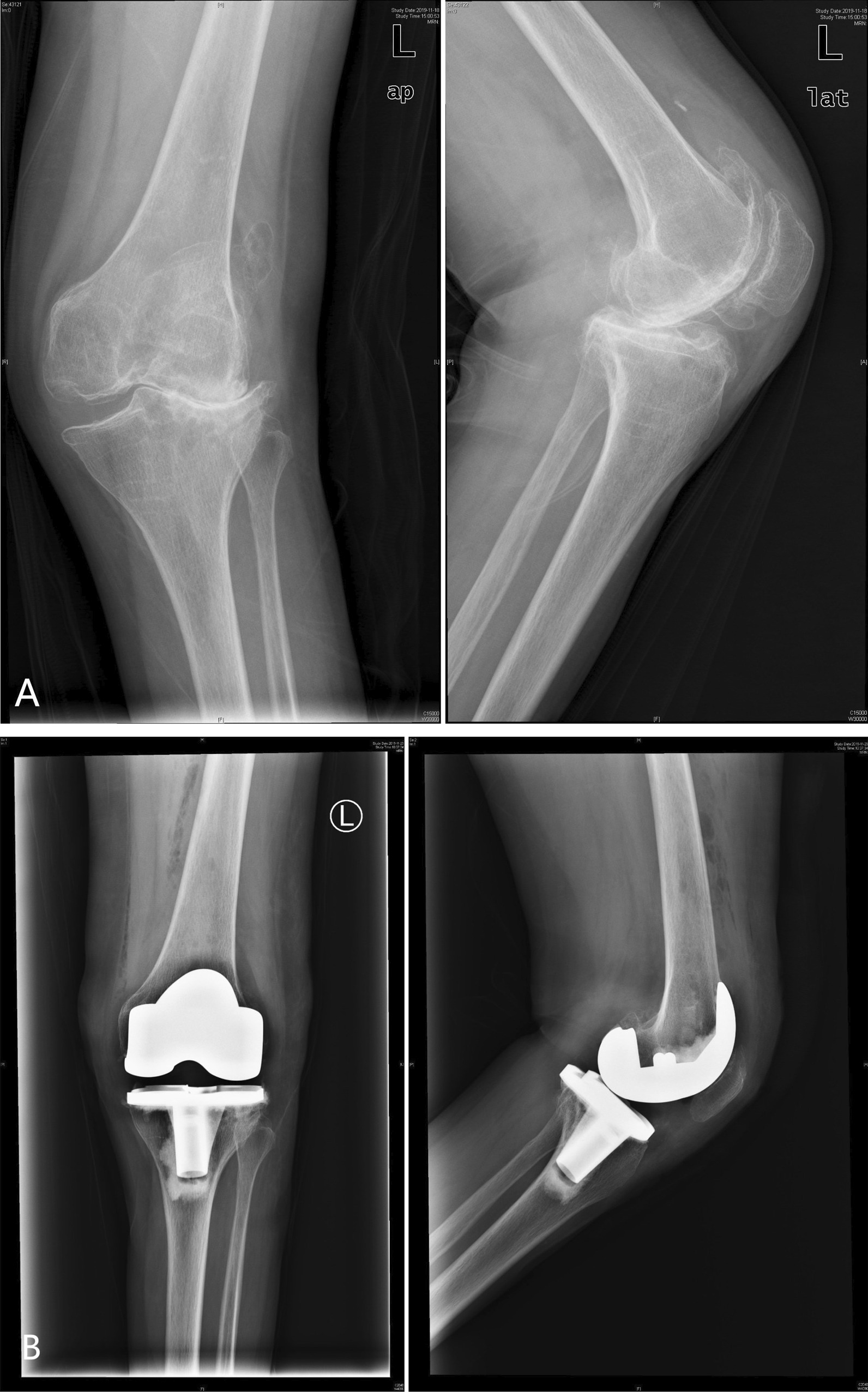
The anteroposterior and lateral knee radiographs of left knee before and after the operation. **A** preoperative valgus knee with osteophyte; **B** postoperative knee status

**Fig. 4:**
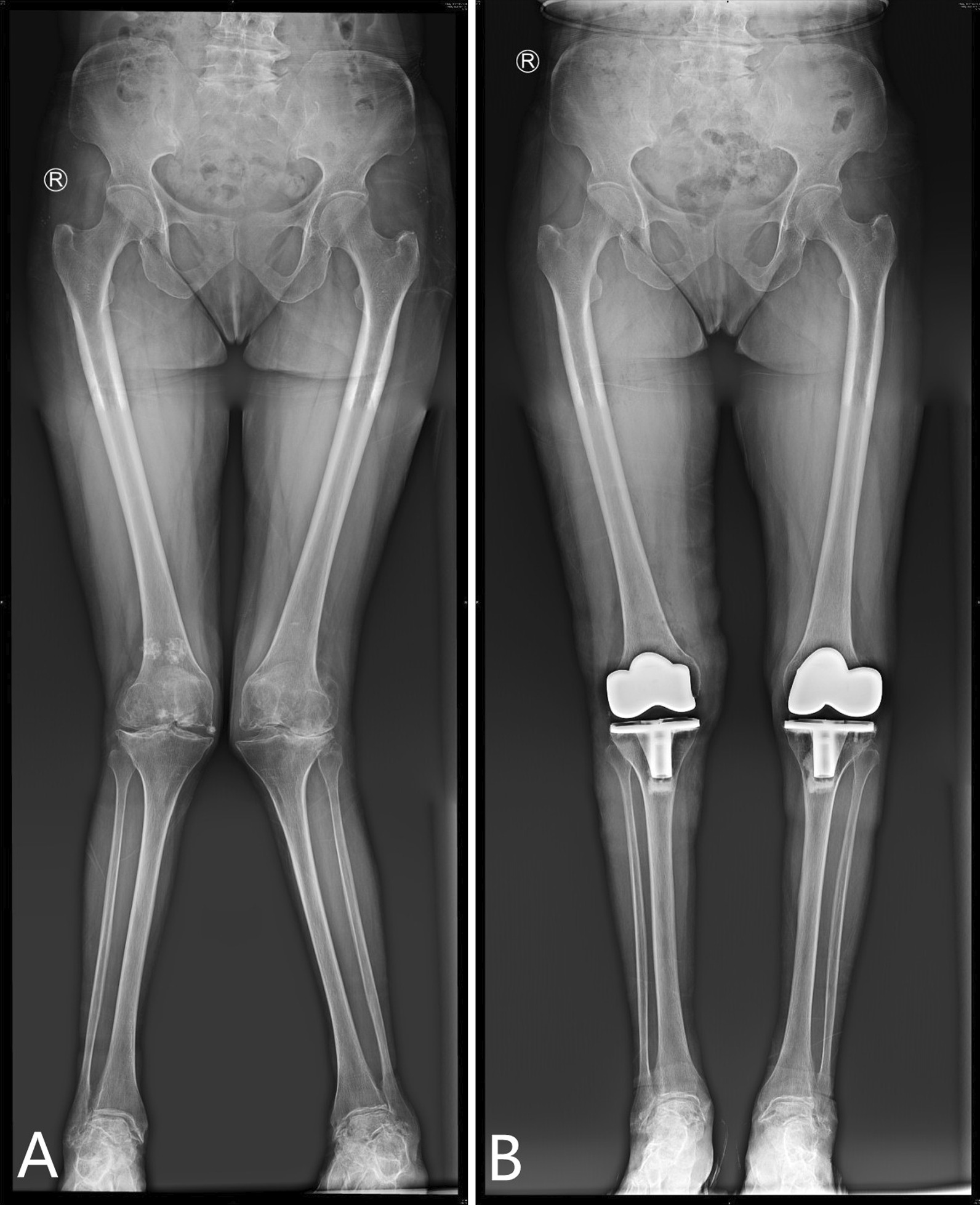
The weight-bearing radiographs of both lower limbs before and after the operation

The SMCO approach was applied in a 56-year-old female patient with bilateral knee valgus. The states of the right and left knees before and after surgery in this patient are shown in Figs. 2 and 3. Figure 4 shows the weight-bearing status of both lower limbs of this patient before and after surgery.

### Complications

During postoperative follow-up, postoperative subluxation of the patella occurred in five cases in the MP group (Table 5). There were no cases of instability, recurrent valgus deformity or radiographic loosening in either group.

**Table 5:** Complications

Variables	SMOC	MP	*P* value
Subluxation of patella	0	5	0.061

## Discussion

Valgus knee was defined as valgus alignment of the anatomical axes of the femur and tibia in the frontal plane exceeding 10° [12]. Pathological changes in the valgus knee involve the bone and soft tissue around the knee. Lateral femoral condyle deficiency, lateral tibial plateau deficiency and external rotation deformity of the tibia are associated with the bone changes seen in the valgus knee. With regard to the soft tissue, contracted lateral tissue and lax medial tissue are typical features of the valgus knee, and are can make surgery difficult. Ranawat [12] classified valgus deformity according to severity, as follows: type I, minimal coronal plane valgus with medial soft tissue stretching; type II, fixed coronal deformity > 10° with attenuated medial soft tissue; and type III, severe bony deformity with impaired medial soft tissue and previous osteotomy.

As the standard approach for TKA, the MP approach is also utilised in the valgus knee. This traditional approach provides good operative exposure and is convenient [14]. Release of the medial structures should be minimised to avoid medial laxity of the knee. However, it is difficult to expose and release the posterolateral joint capsule and soft tissue. Therefore, the MP approach is suitable for mild to moderate valgus knees. Besides, there are numerous literatures reported better exposure with lateral approach. However, this approach also has some problems, such as difficulty in closing the incision. At the same time, almost all doctors are more familiar and are more experienced with the medial approach [15].

The medial subvastus approach has been available for nearly 15 years. Unlike the MP technique, the patella is not everted, and the quadriceps tendon and VMO muscle are not disturbed during medial arthrotomy when using the medial subvastus approach. The medial subvastus approach has also been used in TKA for valgus knees. In a retrospective study of 78 patients (84 knees) undergoing primary TKA for type I or II fixed valgus knees, Koninckx et al. [10] showed that the American Knee Society (AKS) score, function score and flexion improved significantly; alignment was corrected to achieve a hip-knee-ankle (HKA) angle of 181° ± 1.5°, with a postoperative joint line shift of + 2.8 ± 3.2 mm and no clinical instability or osteolytic lines. Shah et al. [16] retrospectively reviewed 112 knees with valgus deformity, and found that the AKS and function scores significantly improved from preoperative mean values of 39 and 36 to 91 and 79, respectively (*P* < 0.001).

However, the subvastus approach sometimes fails to provide adequate exposure [14, 17, 18]. Combining the subvastus approach with limited parapatellar approach, we proposed the SMOC approach, which protects the VMO and patellar tendon from damage or excessive release during exposure. In contrast to the midvastus approach, the SMOC technique involves gradually cutting (0–2-cm-long cut) the VMO tendon insertion from the top medial corner of the patella at an angle of 45°, in the upper lateral instead of upper medial direction. In this way, the moment generated on the VMO facilitates lateral retraction of the patella and decreases the tension on the extensor mechanism and patellar tendon. This results in limited release of tendinous tissue rather than muscle, as well as less pain and blood loss after surgery. The SMOC approach has been used in TKA for varus deformity, but has seldom been applied in TKA for valgus knees.

In this study, three perioperative parameters (amount of drainage, VAS score and time to SLR) and two postoperative parameters (HSS and ROM) were improved in the SMOC group. The MP group had a significantly higher mean drainage amount, and a higher VAS score at 24 h after the operation and time to SLR, compared to the SMOC group. The HSS and ROM were significantly improved in the SMOC group in comparison to the MP group at 1 day, 1 week and 6 weeks, but not at 8 weeks or the last follow-up. These observations suggested that differences in clinical outcomes between MP and SMOC are present only for a short time and are very likely to disappear as early as 6 weeks after the operation. However, this study demonstrated that the SMOC approach can achieve faster recovery after TKA.

Postoperative subluxation of the patella is often encountered after TKA due to lateral femoral condyle deficiency and release of lateral structures of the knee. In this study, however, there were no cases of postoperative subluxation of the patella in the SMOC group, compared to five cases in the MP group. This was likely because the lateral soft tissue was not fully released in the SMOC group, and the extensor mechanism was inadequately reconstructed. The subvastus is the most medial approach for TKA, and the moment needed for patellar displacement is the largest; this minimises the likelihood of patellar dislocation.

## Conclusion

The SMOC approach is suitable for valgus knees, and provides satisfactory recovery without any increase in the incidence of complications.

## Data Availability

The final dataset will be available from the corresponding author.

## Abbreviations

MP: Medial parapatellar approach
SMOC: Subvastus with minimal oblique cut
TKA: Total knee arthroplasty
VAS: Visual analogue scale
ROM: Range of motion
HSS: Hospital for special surgery score
SLR: Straight leg raising
